# Supplementary material for: Modeling glioblastoma heterogeneity as a dynamic network of cell states
Source: Mol Syst Biol. 2021 Sep 16;17(9):e10105. doi: 10.15252/msb.202010105 (PMC8444284; doi:10.15252/msb.202010105)
Supplement: Supplementary file 6 — Source Data for Figure 5 [file MSB-17-e10105-s004.zip › Figure5A_sourcedata/GSEA_3017/hallmarks_stateD.GseaPreranked.1621934542601/HALLMARK_G2M_CHECKPOINT.html]

Details for gene set HALLMARK\_G2M\_CHECKPOINT[GSEA]

|  || Dataset | state23017 |
| Phenotype | NoPhenotypeAvailable |
| Upregulated in class | na\_neg |
| GeneSet | HALLMARK\_G2M\_CHECKPOINT |
| Enrichment Score (ES) | -0.41090453 |
| Normalized Enrichment Score (NES) | -2.1545713 |
| Nominal p-value | 0.0 |
| FDR q-value | 0.006558643 |
| FWER p-Value | 0.012 |
Table: GSEA Results Summary

  

Fig 1: Enrichment plot: HALLMARK\_G2M\_CHECKPOINT      
 Profile of the Running ES Score & Positions of GeneSet Members on the Rank Ordered List

  

| PROBE | GENE SYMBOL | GENE\_TITLE | RANK IN GENE LIST | RANK METRIC SCORE | RUNNING ES | CORE ENRICHMENT || 1 | PTTG1 |  |  | 14 | 0.490 | 0.0020 | No |
| 2 | SQLE |  |  | 25 | 0.421 | 0.0053 | No |
| 3 | MYC |  |  | 77 | 0.313 | -0.0272 | No |
| 4 | HMGB3 |  |  | 80 | 0.311 | -0.0204 | No |
| 5 | MARCKS |  |  | 96 | 0.295 | -0.0245 | No |
| 6 | TROAP |  |  | 109 | 0.283 | -0.0265 | No |
| 7 | BIRC5 |  |  | 130 | 0.271 | -0.0353 | No |
| 8 | TOP1 |  |  | 131 | 0.270 | -0.0280 | No |
| 9 | MKI67 |  |  | 206 | -0.254 | -0.0806 | No |
| 10 | MCM2 |  |  | 226 | -0.256 | -0.0890 | No |
| 11 | NSD2 |  |  | 259 | -0.260 | -0.1077 | No |
| 12 | RBL1 |  |  | 285 | -0.262 | -0.1207 | No |
| 13 | POLA2 |  |  | 312 | -0.264 | -0.1344 | No |
| 14 | HMMR |  |  | 319 | -0.266 | -0.1321 | No |
| 15 | ARID4A |  |  | 448 | -0.279 | -0.2274 | No |
| 16 | CDC25B |  |  | 472 | -0.282 | -0.2383 | No |
| 17 | E2F1 |  |  | 521 | -0.287 | -0.2691 | No |
| 18 | MEIS2 |  |  | 594 | -0.298 | -0.3190 | No |
| 19 | MCM3 |  |  | 625 | -0.305 | -0.3348 | No |
| 20 | PRC1 |  |  | 645 | -0.310 | -0.3417 | No |
| 21 | NUMA1 |  |  | 653 | -0.311 | -0.3390 | No |
| 22 | RAD21 |  |  | 674 | -0.314 | -0.3466 | No |
| 23 | CHAF1A |  |  | 679 | -0.315 | -0.3413 | No |
| 24 | TMPO |  |  | 688 | -0.317 | -0.3391 | No |
| 25 | PLK4 |  |  | 699 | -0.319 | -0.3386 | No |
| 26 | KIF4A |  |  | 706 | -0.320 | -0.3347 | No |
| 27 | PRIM2 |  |  | 764 | -0.336 | -0.3715 | No |
| 28 | CDC20 |  |  | 793 | -0.341 | -0.3848 | No |
| 29 | BRCA2 |  |  | 808 | -0.347 | -0.3867 | No |
| 30 | MAD2L1 |  |  | 819 | -0.350 | -0.3853 | No |
| 31 | BUB3 |  |  | 826 | -0.351 | -0.3806 | No |
| 32 | TFDP1 |  |  | 860 | -0.359 | -0.3974 | No |
| 33 | NEK2 |  |  | 861 | -0.359 | -0.3877 | No |
| 34 | CDKN1B |  |  | 862 | -0.359 | -0.3780 | No |
| 35 | SMC1A |  |  | 885 | -0.367 | -0.3858 | No |
| 36 | STIL |  |  | 888 | -0.369 | -0.3774 | No |
| 37 | SAP30 |  |  | 905 | -0.376 | -0.3801 | No |
| 38 | SLC12A2 |  |  | 911 | -0.379 | -0.3739 | No |
| 39 | INCENP |  |  | 914 | -0.379 | -0.3653 | No |
| 40 | FOXN3 |  |  | 921 | -0.382 | -0.3598 | No |
| 41 | XPO1 |  |  | 967 | -0.400 | -0.3852 | No |
| 42 | CDC45 |  |  | 1000 | -0.413 | -0.3997 | Yes |
| 43 | KIF11 |  |  | 1003 | -0.416 | -0.3901 | Yes |
| 44 | CDC6 |  |  | 1008 | -0.418 | -0.3820 | Yes |
| 45 | POLQ |  |  | 1033 | -0.436 | -0.3896 | Yes |
| 46 | TPX2 |  |  | 1034 | -0.437 | -0.3778 | Yes |
| 47 | KIF22 |  |  | 1051 | -0.446 | -0.3786 | Yes |
| 48 | SMC2 |  |  | 1062 | -0.452 | -0.3744 | Yes |
| 49 | CDKN2C |  |  | 1067 | -0.454 | -0.3654 | Yes |
| 50 | EZH2 |  |  | 1070 | -0.455 | -0.3547 | Yes |
| 51 | PLK1 |  |  | 1082 | -0.460 | -0.3511 | Yes |
| 52 | RACGAP1 |  |  | 1112 | -0.479 | -0.3615 | Yes |
| 53 | BARD1 |  |  | 1117 | -0.486 | -0.3515 | Yes |
| 54 | TTK |  |  | 1119 | -0.487 | -0.3392 | Yes |
| 55 | NUSAP1 |  |  | 1154 | -0.515 | -0.3526 | Yes |
| 56 | DBF4 |  |  | 1157 | -0.520 | -0.3402 | Yes |
| 57 | CCNF |  |  | 1160 | -0.522 | -0.3277 | Yes |
| 58 | KNL1 |  |  | 1167 | -0.534 | -0.3181 | Yes |
| 59 | ORC6 |  |  | 1179 | -0.551 | -0.3121 | Yes |
| 60 | KIF2C |  |  | 1183 | -0.558 | -0.2994 | Yes |
| 61 | CKS1B |  |  | 1198 | -0.577 | -0.2951 | Yes |
| 62 | KIF15 |  |  | 1213 | -0.610 | -0.2899 | Yes |
| 63 | SMC4 |  |  | 1224 | -0.638 | -0.2807 | Yes |
| 64 | MYBL2 |  |  | 1225 | -0.639 | -0.2634 | Yes |
| 65 | CKS2 |  |  | 1226 | -0.641 | -0.2461 | Yes |
| 66 | CENPA |  |  | 1243 | -0.679 | -0.2406 | Yes |
| 67 | AURKB |  |  | 1250 | -0.700 | -0.2265 | Yes |
| 68 | CCNA2 |  |  | 1255 | -0.716 | -0.2104 | Yes |
| 69 | KIF23 |  |  | 1265 | -0.741 | -0.1976 | Yes |
| 70 | TACC3 |  |  | 1268 | -0.751 | -0.1789 | Yes |
| 71 | AURKA |  |  | 1271 | -0.763 | -0.1599 | Yes |
| 72 | NDC80 |  |  | 1278 | -0.810 | -0.1429 | Yes |
| 73 | KPNA2 |  |  | 1297 | -0.972 | -0.1311 | Yes |
| 74 | FBXO5 |  |  | 1301 | -0.992 | -0.1067 | Yes |
| 75 | CDK1 |  |  | 1311 | -1.433 | -0.0752 | Yes |
| 76 | UBE2C |  |  | 1312 | -1.466 | -0.0356 | Yes |
| 77 | TOP2A |  |  | 1313 | -1.528 | 0.0056 | Yes |
Table: GSEA details [plain text format]

  

Fig 2: HALLMARK\_G2M\_CHECKPOINT: Random ES distribution      
 Gene set null distribution of ES for **HALLMARK\_G2M\_CHECKPOINT**
